# Methods and validity indicators for measuring adherence and persistence to aspirin in secondary cardiovascular prevention: a systematic review

**DOI:** 10.3389/fcvm.2025.1570331

**Published:** 2025-05-26

**Authors:** Rauf Nouni-García, Elisabeth Ramirez-Familia, Adriana López-Pineda, Amanda Esquerdo-Arroyo, Álvaro Carbonell-Soliva, María Martínez-Muñóz, Antonio F. Compañ Rosique, Jose A. Quesada, Concepción Carratalá-Munuera, Vicente F. Gil-Guillén

**Affiliations:** ^1^ Pathology and Surgery Department, Medical School, University Miguel Hernández de Elche, Alicante, Spain; ^2^ Institute of Health and Biomedical Research of Alicante, General University Hospital of Alicante, Diagnostic Center, Alicante, Spain; ^3^ Network for Research on Chronicity, Primary Care and Health Promotion (RICAPPS), San Juan de Alicante, Spain; ^4^ Primary Care Research Center, Miguel Hernandez University, San Juan de Alicante, Spain; ^5^ Department of Clinical Medicine, University of Miguel Hernández Elche, Comunidad Valenciana, Spain; ^6^ Center for Research on Aging, Elche, Spain

**Keywords:** aspirin, cardiovascular disease, secondary prevention, medication adherence, platelet function tests

## Abstract

**Background:**

Aspirin (acetylsalicylic acid, ASA) is widely recommended for long-term secondary cardiovascular prevention (SCP), but its clinical effectiveness depends on patient adherence, which remains suboptimal. Understanding how adherence and persistence to ASA are measured is essential to improving outcomes. This systematic review aimed to identify the methods used to assess adherence and persistence to ASA in SCP and evaluate their validity indicators.

**Methods:**

We systematically searched EMBASE, MEDLINE, and Scopus for studies published up to October 30, 2023, reporting methods for measuring adherence or persistence to ASA in adults undergoing secondary cardiovascular prevention. Two reviewers independently screened articles and extracted data on study characteristics, measurement methods, and validity indicators. The results were synthesized in tabular form according to method type (indirect or direct) and outcome assessed (adherence or persistence). Risk of bias was evaluated for studies that conducted validation analyses of the measurement methods.

**Results:**

Forty studies were included, most conducted in the United States. Indirect methods predominated: self-report questionnaires (45%, *n* = 18) and pharmacy dispensing records (32.5%, *n* = 13) were the most common tools for assessing adherence. Direct methods, such as platelet function tests or biochemical assays, were less frequently used (25%, *n* = 10). For persistence, dispensing records were the most used method (70%, *n* = 7). No indirect method reported validation specifically for ASA adherence or persistence. Validity indicators were only partially available for some direct methods.

**Conclusions:**

Adherence and persistence to ASA in SCP are primarily measured through indirect methods, with a lack of specific validation for ASA. There is a critical need to develop standardized, validated tools that integrate both direct and indirect measures and address gender-specific barriers to adherence.

**Systematic Review Registration:**

https://www.crd.york.ac.uk/PROSPERO/view/CRD42023470993, PROSPERO identifier CRD42023470993.

## Background

Cardiovascular diseases (CVD) remain one of the leading causes of mortality worldwide. These diseases account for 19.7 million deaths annually across the globe, according to the World Health Organization, and it is expected that, due to population ageing, this number will continue to rise in the coming decades (1). In Spain, CVD was the second leading cause of death, with a mortality rate of 237.5 deaths per 100,000 inhabitants (26.5%), in 2023 (2). Effective secondary cardiovascular prevention (SCP), through lifestyle and pharmacological interventions, reduces mortality, prevents recurrent events, and improves quality of life (3). Among the recommended medications, acetylsalicylic acid (ASA), commonly referred to as aspirin, is an antiplatelet agent that works by inhibiting thromboxane A2 formation, thereby reducing platelet aggregation and preventing vascular obstruction due to clot formation (4). Long-term low-dose aspirin use has been shown to significantly reduce the risk of both fatal and non-fatal cardiovascular events, as well as the number of hospitalization days, in high-risk individuals undergoing SCP, regardless of age or sex (5–8). Its use is recommended with a Class I, Level A indication (7).

The effectiveness of aspirin therapy is closely linked to patient adherence. Medication adherence is defined as the extent to which a patient's medication-taking behavior corresponds with agreed recommendations from a healthcare professional. Despite its importance, only 50% of chronic patients in developed countries adhere to their treatment regimens, with even lower adherence rates reported in developing countries (9). Medication adherence is a multifactorial phenomenon determined by five interrelated domains: patient characteristics, social and family environment, disease characteristics, therapeutic regimen, and healthcare system conditions (10–15). Since the effectiveness of treatment depends not only on daily intake but also on maintaining therapy over time, another concept, persistence, has emerged in the context of medication adherence. Persistence is defined as the period between treatment initiation and the last dose taken before treatment discontinuation, or the proportion of days a patient continues the treatment over a given period (16). It is measured either as the proportion of days covered (PDC) by the medication or as the mean number of days until treatment discontinuation (17). In the case of ASA, a cohort study conducted in the Netherlands showed that the proportion of persistent users dropped from 77.3% after one year of follow-up to 27.5% after ten years (18). Lack of continuity compromises treatment effectiveness, preventing the expected benefits of the medication and increasing the risk of recurrence.

Adherence measurement methods can be classified as direct or indirect. Direct methods, such as directly observed therapy, therapeutic drug monitoring, and ingestible sensor systems, are objective, specific, and highly accurate but are often costly and impractical for routine clinical practice. Indirect methods include patient self-report questionnaires, pill counts, the proportion of days covered (PDC), the medication possession ratio (MPR), and medication event monitoring systems (MEMS), among others (19–21). Among these, self-report questionnaires are widely used due to their simplicity, practicality, and low cost; however, they have notable limitations, including subjectivity, recall bias, and response bias, as the information is provided by the patient. Commonly used questionnaires include the Haynes-Sackett Test, the Morisky-Green Test (22, 23), and the MMA-S 8 questionnaire (17, 24–26), the latter being one of the most commonly employed in clinical practice. Despite its validation in other populations and pathologies (27–29), the MMA-S 8 questionnaire has been used in unvalidated clinical settings, proving unsuitable for certain populations, such as patients with type 2 diabetes in Spain (30). Moreover, the results of the original MMA-S 8 study were recently retracted due to inconsistencies in its sensitivity and specificity values (26).

It is crucial for healthcare professionals to be familiar with validated tools for measuring adherence to ASA, given the severe consequences of non-adherence in chronic diseases such as CVD. Failure to identify non-adherence as the cause of poor disease control may result in a medication being incorrectly deemed ineffective. This can lead to unnecessary treatment intensification, unwarranted diagnostic tests, or even misinterpretation of clinical trial results when medication adherence is unknown (31). The objective of this systematic review was to identify the methods used in research to measure adherence and persistence to ASA in patients undergoing secondary cardiovascular prevention, as well as to assess the validity and accuracy of these methods.

## Methods

The protocol for this systematic review was registered in PROSPERO (ID: CRD42023470993). The PRISMA guidelines and checklist (32) were followed to report the methodology and results. This study was approved by the Office of Responsible Research at Miguel Hernández University in Spain (TFG.GME.ALP.ERF.231213).

### Eligibility criteria

Studies were eligible for this review if they reported measuring adherence and/or persistence to aspirin treatment in patients undergoing SCP and mentioned the specific measurement method used. Studies that assessed adherence or persistence to ASA alongside other medications were excluded. The study population included individuals aged 18 years or older undergoing SCP for conditions such as ischemic heart disease, acute myocardial infarction, stroke, cerebral hemorrhage, transient ischemic attack, renal failure, heart failure, peripheral artery disease, aortic dissection, or diabetic and hypertensive retinopathy, all treated with ASA. Eligible study designs encompassed observational (cross-sectional, case-control, and cohort) and experimental studies. Excluded were letters, editorials, clinical cases, reviews, opinion articles, conference abstracts, study protocols, and non-scientific studies, as well as those written in a non-Latin alphabet. There were no restrictions on the publication date.

### Information sources and search strategy

The search was conducted in three databases: EMBASE, MEDLINE, and Scopus. All studies published from the inception of each database up to October 30, 2023, were included. No additional searches were performed.

The search strategy included key terms such as “Treatment Adherence and Compliance”, “Medication Adherence”, “Aspirin”, “Cardiovascular Diseases”, and “Acute Coronary Syndrome”, combining controlled vocabulary and free-text terms with Boolean operators. Filters were applied to restrict the results by study type and population age. Details of the search strategy for each database are provided in Supplementary Material 1.

### Selection process

Articles retrieved from the database searches were exported to the Rayyan® platform. Duplicates were manually removed after automatic detection using Rayyan. Subsequently, the selection process was conducted by six independent reviewers, who divided the workload based on study type. Two pairs of reviewers screened the titles and abstracts of observational studies and experimental studies, respectively, during the first selection round. In the second round, the same pairs independently assessed the eligibility of the studies included in the first round by reviewing the full texts. Any discrepancies or conflicts in both rounds were resolved by a third reviewer assigned to each group. The reviewers responsible for study selection included researchers with experience in systematic reviews and expertise in cardiovascular prevention and adherence research, as well as two supervised student researcher. For studies without free full-text access, interlibrary loan requests were submitted to Miguel Hernández University. Studies that could not be retrieved were excluded.

### Data extraction process

Data extraction from the finally included articles was performed by two researchers, with another researcher reviewing the extracted data for accuracy. The reviewers responsible for data extraction included researchers with experience in systematic reviews and expertise in cardiovascular prevention and adherence research. The extracted information included the principal author, year of publication, study location, study design, study population, and whether adherence, persistence, or both were measured. Adherence was defined as the extent to which a patient's medication-taking behavior corresponds with the prescribed dosage regimen, while persistence was defined as the duration of continuous medication use without interruption. These definitions follow the ABC taxonomy framework (16). Additional extracted data included sample size, study setting, methods used to measure adherence or persistence (type and description), criteria for classifying patients as adherent/persistent or non-adherent/non-persistent, validity indicators of the measurement methods (if available), and psychometric properties of the questionnaires (if available). Validity indicators were considered classical measures of method accuracy, such as sensitivity, specificity, positive predictive value, and negative predictive value. For self-report questionnaires, psychometric properties (e.g., internal consistency assessed through Cronbach's alpha, test–retest reliability, and construct validity) were extracted separately, recognizing their conceptual distinction from classical validity indicators.

### Synthesis of results

A descriptive synthesis of the characteristics of the included studies was performed. Additionally, a narrative synthesis was conducted to describe separately the methods used to measure adherence and persistence, including a tabulation of the validity indicators for methods validated specifically for ASA and the psychometric properties of the questionnaires used. Due to the scarcity of studies providing validity indicators for measuring adherence and persistence to ASA, a meta-analysis could not be performed.

### Risk of bias assessment

A formal risk of bias (RoB) assessment was not conducted for all included studies, as the primary objective of this systematic review was not to evaluate the effectiveness of interventions or the magnitude of adherence or persistence outcomes (which was the main objective of most included studies), but rather to identify and describe the methods used to measure adherence and persistence to aspirin (regardless of the studies’ primary aims) and to collect available information on their validation. In the majority of included studies, adherence or persistence was reported as a secondary outcome or a descriptive measure, and the studies did not aim to critically assess or validate the measurement tools used. In such cases, applying traditional RoB tools would primarily reflect the quality of the main research objective (e.g., intervention effectiveness, adherence or persistence outcome), not the reliability of the measurement method itself, which is not the focus of this review and may lead to misinterpretation regarding the quality of the adherence (or persistence) measurement methods themselves.

However, a risk of bias assessment was conducted for the subset of studies whose primary objective was to validate or evaluate a method for measuring adherence or persistence to aspirin, particularly those employing direct measurement approaches, such as platelet function tests or biochemical assays. In these cases, the quality of the study is directly relevant to the reliability of the reported validation indicators. The QUADAS-2 tool was used for diagnostic accuracy studies, and the COSMIN Risk of Bias checklist was applied to studies evaluating the psychometric properties of questionnaires.

## Results

**Figure 1:**
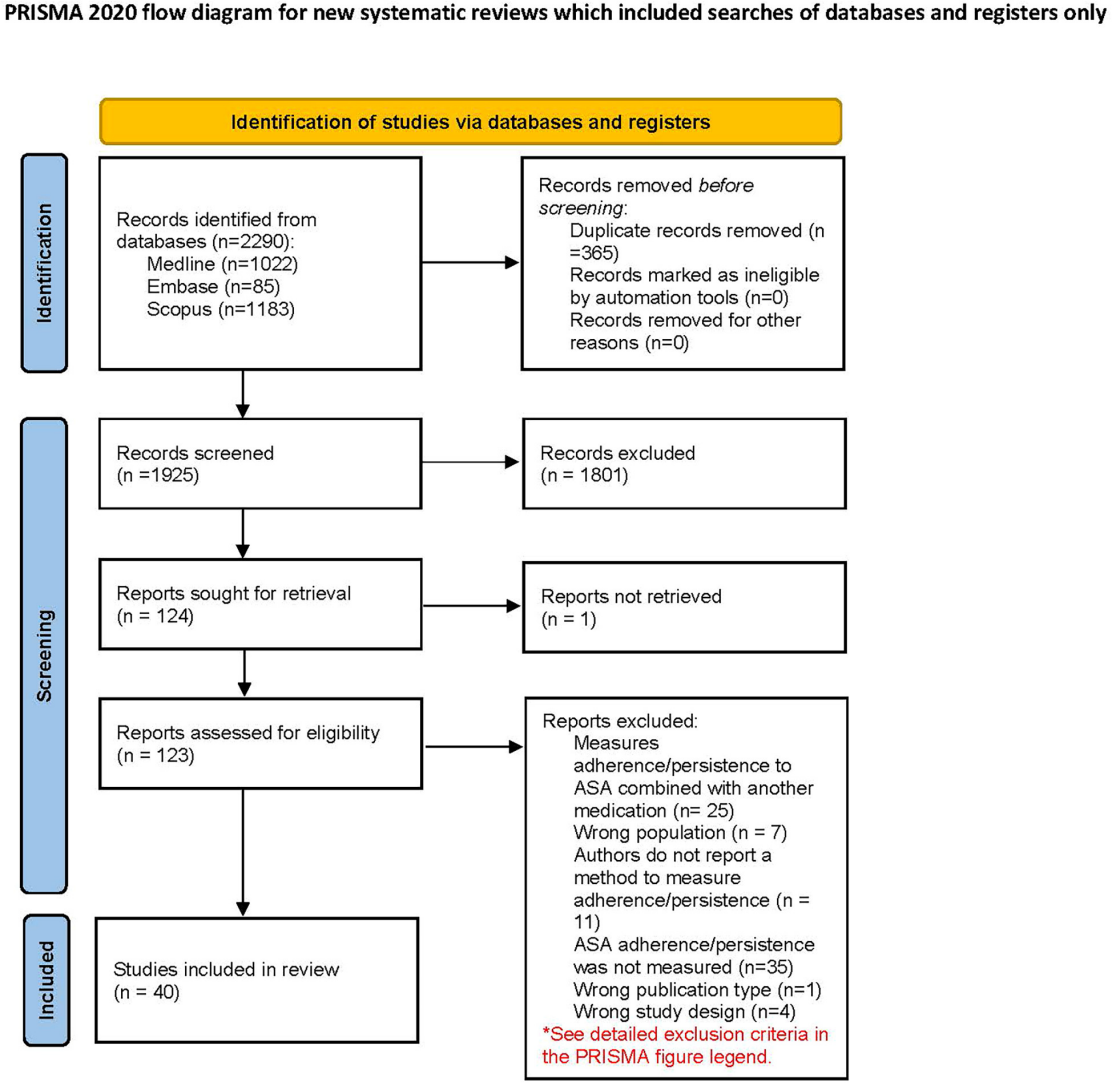
PRISMA flow diagram. *Reasons for exclusion included: combining ASA adherence with other medications (e.g., dual antiplatelet therapy), studies focused on non-SCP populations (e.g., primary prevention or non-cardiovascular conditions), lack of adherence measurement or unclear methodology (e.g., adherence mentioned but not described), and studies with designs not meeting inclusion criteria (e.g., case reports, narrative reviews). Adapted with permission from “PRISMA 2020 flow diagram template for systematic reviews” by Page et al., licensed under CC BY 4.0.

A total of 2,290 articles were identified through the search, and 1,925 titles and abstracts were screened after duplicate removal. Subsequently, the full texts of 123 studies were evaluated, resulting in the inclusion of 40 articles in the review. The main reason for exclusion was the absence of adherence (or persistence) measurements for ASA. Figure 1 presents the PRISMA flow diagram (32), which details the study selection process throughout the systematic review. Two studies by García Rodríguez et al. (33, 34), included in this review, were conducted with different objectives but focused on the same population. Both studies employed the same method to measure persistence and applied identical criteria to define non-persistence. As a result, they were combined and treated as a single outcome, with only one method extracted instead of two.

### Characteristics of included studies

Table 1 shows the characteristics of the included articles, which were published between 2002 and 2023. Most of the studies were conducted in the United States (*n* = 15), followed by the United Kingdom (*n* = 4) and China (*n* = 3). Regarding study types, the majority were cohort studies (*n* = 22), followed by experimental studies (*n* = 13) and cross-sectional studies (*n* = 3). Sample sizes varied widely, ranging from 14 to 335,624 participants. Concerning the study setting, most were conducted in hospital settings (*n* = 15), while others were carried out in hospital-based outpatient follow-up settings (*n* = 9), outpatient-only settings (*n* = 8), and clinical research centers (*n* = 6). A large proportion of the articles (72.5%) focused on adherence (*n* = 29), whereas fewer studies measured persistence (5%, *n* = 2) or both adherence and persistence (22.5%, *n* = 9).

**Table 1:** Descriptive characteristics of included studies (*n* = 40).

Author, year of publication	Study location	Study design	Study population	Study setting	Sample size
Bhasin V, 2020 (72)	USA	Cross-sectional	Adults with a history of MI.	Outpatient	2,173
Bi, Y, 2009 (73)	China	Cohort	Patients with STEMI, NSTEMI, or UA.	Hospital	2,901
Cao, J, 2023 (19)	China	Cohort	Patients with CHD treated with antiplatelet therapy one year post-discharge.	Hospital	289
Castellano JM, 2014 (38)	Argentina, Brazil, Italy, Paraguay, Spain	RCT	Men and women over 40 years old with a history of MI within the past two years.	Outpatient	2,118
Chow, CK, 2024 (74)	Australia	RCT (multicentre)	Patients diagnosed with ACS (MI or unplanned coronary ischaemia due to prior or revascularized (CHD).	Hospital	1,424
Cotter, G, 2004 (50)	USA and Israel	Cohort	Patients with MI treated with 100 mg/day aspirin for at least one month before inclusion.	Outpatient	73
Dangas G.D, 2013 (75)	USA	RCT	Patients with STEMI meeting criteria for prolonged DAPT after PCI with stent implantation.	Hospital	2,997
Flynn R, 2012 (41)	Scotland	Cohort	Patients with a first-ever ischemic stroke	Hospital	1,407
García Rodríguez, L.A, 2011 (33, 34)	UK	Cohort	Adults aged 50–84 years with a history of cardiovascular or cerebrovascular events, treated with low-dose aspirin.	Outpatient	39,512
Halvorsen S, 2016 (21)	Norway	Cohort	Patients under 85 years old with MI, followed for 24 months or until death.	Hospital	42,707
Johnson, C, 2012 (76)	New Zealand	Cohort	Patients with documented ischaemic stroke in an observational cohort study.	Outpatient	51
Komiya T, 1994 (49)	Japan	RCT	Patients with a history of transient ischaemic attacks or cerebral thrombosis.	Hospital with outpatient follow-up	61
Kronish IM, 2010 (77)	USA	Cohort	Patients with ACS (first episode of UA or MI), treated in hospital and followed as outpatients.	Hospital with outpatient follow-up	105
Kronish, IM, 2014 (78)	USA	Cohort	Patients with MI or UA with BDS scores <5 or ≥10.	Hospital	169
Kulkarni, 2006 (79)	USA	Cohort	Patients with significant CAD (≥70% occlusion) documented by cardiac catheterisation.	Hospital	1,326
Lago A, 2006 (51)	Spain	Cross-sectional	Patients with ischaemic stroke treated with aspirin for at least six months.	Outpatient	73
Maddison R, 2021 (80)	New Zealand	RCT	Adults with ACS undergoing PCI, clinically stable and able to provide informed consent.	Hospital	306
Martin-Latry, K, 2022 (42)	France	Cohort	Patients with MI documented in registries, followed for one year.	Hospital with outpatient follow-up	3,015
Mortensen, J, 2008 (20)	Denmark	Cohort	Patients divided into groups with stable CAD treated with aspirin and CAD documented via angiography.	Not specified	64
Newby KL, 2006 (81)	USA	Cohort	Patients with CADV (at least 1 documented coronary stenosis of 50% or coronary bypass surgery) who had at least 1 follow-up survey completed during the period 1995 to 2002.	Hospital	31,750
O'Carroll R, 2011 (55)	UK	Cohort	Patients with ischaemic stroke recruited one year post-event.	Hospital	180
Östbring MJ, 2021 (43)	Sweden	RCT	Patients with angiographically confirmed CHD treated in an outpatient setting.	Outpatient	316
Rahhal, A, 2021 (82)	Qatar	Cohort	Patients with STEMI undergoing primary angioplasty, followed for 10 months.	Hospital with outpatient follow-up	1,334
Rieckman, N, 2006 (83)	USA	Cohort	Patients with MI or UA treated with aspirin at doses of 81 mg/day or 325 mg/day, followed as outpatients.	Hospital with outpatient follow-up	165
Rieckman, N, 2006 (84)	USA	Cohort	Patients with MI or UA treated with aspirin at doses of 81 mg/day or 325 mg/day, followed as outpatients.	Hospital with outpatient follow-up	165
Riegel, B, 2020 (85)	USA	RCT	Patients with ACS discharged with a prescription for aspirin and self-managing their medication using a smartphone.	Hospital	130
Riess H, 1986 (52)	Germany	RCT	Patients undergoing PTCA in a clinical research centre.	Clinical research centre	14
Rinfret S, 2013 (45)	Canada	RCT	Patients who received DES with prescribed aspirin and clopidogrel in a clinical setting.	Clinical research centre	300
Robinson J.G., 2010 (46)	USA	Post-hoc analysis of clinical trials	Postmenopausal women aged 50–79 years with documented CHD.	Clinical research centre	2,627
Rubak P, 2013 (56)	Denmark	Experimental validation study	Patients with stable CHD and healthy volunteers in a clinical setting.	Clinical research centre	60
Schwartz KA, 2005 (86)	USA	Cross-sectional	Adults with a history of MI treated with aspirin for at least one month.	Hospital	190
Shemesh E, 2006 (53)	USA and Israel	Prospective observational	Patients with MI followed up in a clinical research setting.	Clinical research centre	65
Simpson E, 2003 (40)	Canada	Cohort	Patients over 65 years old with MI documented in historical records.	Hospital with outpatient follow-up	14,057
The Coronary Drug Project Research Group, 1976 (47)	USA	RCT	Men with confirmed history of MI.	Clinical research centre	1,529
Vora, P, 2022 (44)	Germany and the UK	Cohort	Patients with CVD, coronary bypass, or PCI, treated with at least two prescriptions of aspirin for secondary prevention.	Database/Outpatient	335,624
Wei, L, 2008 (87)	Scotland	Cohort	Patients with documented CVD followed for at least 180 days after their first aspirin or statin prescription.	Hospital with outpatient follow-up	7,657
Wheat, H, 2021 (88)	USA	Cohort	Patients with atherosclerotic disease or systolic HF in NYHA class II or III, clinically followed.	Hospital	151
Yasmina, A, 2017 (18)	Netherlands	Cohort	Patients with MI registered in databases and followed as outpatients.	Hospital with outpatient follow-up	4,690
Zhong J, 2023 (37)	China	Cohort	Patients with ischaemic stroke treated in hospital with outpatient follow-up.	Hospital with outpatient follow-up	229

ACS, acute coronary syndrome; AHA, American Heart Association; AAS, acetylsalicylic acid; BDS, beck depression scale; CAD, coronary artery disease; CHD, coronary heart disease; CVD, cardiovascular disease; DES, drug-eluting stent; DAPT, dual antiplatelet therapy; HF, heart failure; MI, myocardial infarction; NSTEMI, non-ST-elevation myocardial infarction; NYHA, New York Heart Association Functional Classification; PCI, percutaneous coronary intervention; PTCA, percutaneous transluminal coronary angioplasty; RCT, randomised controlled trial; STEMI, ST-elevation myocardial infarction; UA, unstable angina.

## Measurement methods identified

### Indirect methods

#### Measurement of adherence

**Figure 2:**
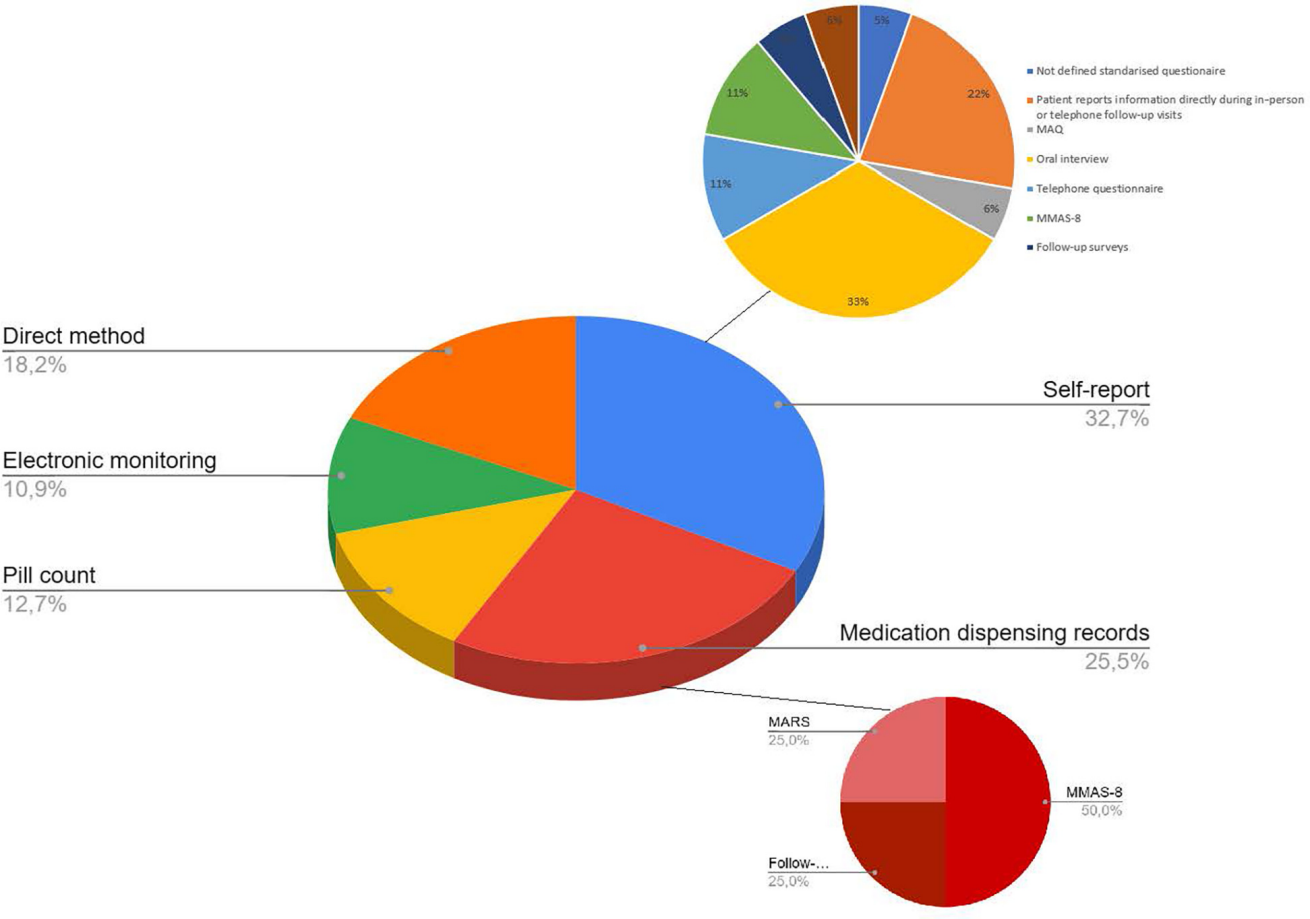
Pie chart illustrating the proportional distribution of aspirin adherence measurement methods reported in this review. MARS, Medication Adherence Rating Scale; MAQ, Medication Adherence Questionnaire; MMAS, Morisky Medication Adherence Scale*.*

Table 2 shows the methods used to measure adherence to ASA in the reviewed studies, including definitions of adherent patients and validation information, if available. These methods, listed in descending order of frequency, included self-report tools (*n* = 18), medication dispensing records (*n* = 13), direct methods (*n* = 10) involving blood-based platelet function tests (*n* = 7) or ASA/metabolite detection (*n* = 3), MEMS (*n* = 6), and pill count methods (*n* = 7). Figure 2 illustrates the percentage of studies using each type and subtype of methods classified for measuring adherence to aspirin. The following sections describe each type of method in detail.

1. Self-report methods
  • Direct patient-reported adherence assessment via medication recall and confirmation (in person or by phone): Patients were asked to list their medications. If their answers matched the prescribed medications at discharge, they were considered adherent. Alternatively, they were directly asked whether they were taking their medications as instructed.
  • Morisky 8-item Adherence Scale (MMAS-8) (26): This scale consists of eight items, with a total score ranging from 0 to 8, where lower scores indicate higher adherence.
  • Morisky 4-item Adherence Scale (MMAS-4, MAQ) (35): This scale consists of four items, with a total score ranging from 0 to 4, where lower scores indicate higher adherence.
  • Medication Adherence Report Scale (MARS) (36): This is a five-item scale rated on a five-point scale from 1 (“very often”) to 5 (“never”). The total score ranges from 5 to 25, with higher scores indicating better adherence.
  • Pill count via self-report (37): Patients were considered adherent if they took 100% of their prescribed pills. Partial adherence was defined as taking more than 75% but less than 100%, while severe non-adherence was defined as taking 75% or fewer of the prescribed pills.
2. Pill count methods

**Table 2:** Methods reported by studies for measuring adherence and/or persistence (*n* = 40).

Author and year of publication	Adherence/persistence	Measurement method	Definition of adherent/persistent patient	Validation	Validity indicators	Psychometric properties of questionnaire
Bhasin V, 2020 (72)	Adherence	**Self-report: p**atients were asked about aspirin intake daily or alternate-day.	Adherent patients were those who answered “yes” to taking aspirin regularly	No	No	–
Bi, Y, 2009 (73)	Adherence	**Self-report:** standardised forms(undefined)completed via telephone or in-person at discharge and at 6 and 12 months follow-up	Adherent if continuing with all four medications simultaneously at 6 and 12 months post-discharge.	No	No	–
Cao, J, 2023 (19)	Adherence	**Self-report:** telephone follow-ups and outpatient visits.	Telephone follow-up and outpatient visits. Undefined. It appears to complement the PDC.	No		
		**Medication dispensing records:** PDC	Adherent patient if PDC ≥ 80%.	No		
Castellano JM, 2014 (38)	Adherence	**Self-report:** MAQ (4 items).	MAQ score ≤16 identifies good adherence.	Validated by other authors for hypertensive patients (35)	Sensitivity: 81%, Specificity: 44%	Internal consistency: 0.61
		**Pill count.**	A pill count between 80% and 110% was considered good adherence.	No	–	–
Chow, CK, 2024 (74)	Adherence	**Self-report:** oral interview about aspirin intake	Adherent (self-reported): proportion of prescribed medications taken >80% (> 24/30 days in the previous month) at 6 and 12 months unless contraindicated.	No	–	–
		**Medication dispensing records:** Administrative records from the Pharmaceutical Benefits Scheme (PBS) were analyzed to validate adherence**.**	Adherent (dispensed records): at least 5 months of medication supplied within each period (0–6 months and 6–12 months).			
Cotter, G, 2004 (50)	Adherence	**Direct method:** thromboxane B2 (TxB2) levels within the range observed in untreated patients.	Adherence: Patients with TxB2 levels within the range observed in those taking aspirin (≤0.5 nmol/10^11 platelets).	No	No	–
		**Self-report:** structured interview about aspirin intake.	Adherent if taking medication more than half of the time. Non-adherent patient is one who reveals not taking it.			
Dangas GD, 2013 (75)	Adherence	**Self-report:** oral interview about aspirin intake.	Adherent: complied with the prescribed treatment throughout the follow-up period.	No	–	–
Flynn R, 2012 (41)	Adherence	**Medication dispensing records:** PDC.	Adherence if patients received a continuity of supply within the period of persistence.	No	–	–
	Persistence	**Medication dispensing records:** PDC.	Persistence if patients continued their regimen without a gap of more than 28 days.	No	–	
García Rodríguez, L.A, 2011 (33, 34)	Persistence	**Medication dispensing records:** PDC.	Interruption defined as no prescription renewal >30 days.	No	No	–
Halvorsen S, 2016 (21)	Adherence	**Medication dispensing records:** MPR.	Adherent: no significant interruptions in medication collection from the pharmacy 12 months before AMI up to 24 months after.	No	No	–
Johnson C, 2012 (76)	Adherence	**Self-report:** telephone questionnaireabout medication intake.	Adherent: reported medication list matched the prescribed medications at discharge.	No	No	–
Komiya T, 1994 (49)	Adherence	**Self-report:** oral interview about aspirin intake**.**	Adherent if patient reported taking medication as prescribed	No	–	–
		**Pill count**	Non defined	No	–	–
		**Direct method:** platelet aggregation measurements using blood samples before and after treatment administration.	Laboratory tests confirmed adequate platelet aggregation inhibition. Diagnostic threshold: maximum aggregation rate reduction <60% with 2 µg/mL collagen.	Exploratory internal validation: platelet aggregation testing with defined thresholds and repeat testing; classification of adherence based on results.	Thresholds for adherence were defined based on a reduction of maximum aggregation to <60% (collagen concentration of 2 µg/mL for aspirin and ADP of 2 µmol/L for ticlopidine). Repeated tests in uncertain cases.	–
Kronish IM, 2010 (77)	Adherence	**Electronic monitoring:** MEMS.	Adherent: >80%	Validated by other authors in hypertensive patients. (39)	Sensitivity: 76%; specificity: 83%, Correlation coefficient: 0.20.	–
Kronish IM, 2014 (78)	Adherence	**Electronic monitoring:** MEMS.	Adherent if open pill bottle once daily for ≥80% of days during 3 months of follow-up.	Validated by other authors in hypertensive patients. (39)	Sensitivity: 76%, Specificity: 83%, Correlation coefficient: 0.20.	–
Kulkarni, 2006 (79)	Adherence	**Self-report**: telephone interview using a structured questionnaire about aspirin intake	Adherent if continued regimen at 12 months similar to that recommended at hospital discharge, evaluated individually for each medication class and as a composite.	No	No	–
Lago A, 2006 (51)	Adherence	**Self-report:** oral interview about aspirin intake.	Non-defined	No	–	–
		**Direct method:** thromboxane A2 (TxA2) synthesis in blood samples using ELISA technique.	Adherent if Tb-A2 inhibition was ≥80% compared to untreated individuals	No	–	–
Maddison R, 2021 (80)	Adherence	**Dispensing records:** MPR.	MPR ≥80%	No	–	–
		**Self-report:** MMAS-8.	MMAS-8: (0 = high, 1–2 = medium and 3–6 = low).	Validated by previous authors for hypertensive patients (results retracted) (26)	Sensitivity: 93%, Specificity: 53%.	Cronbach's alpha: 0.83.
Martin-Latry, K, 2022 (42)	Adherence	**Medication dispensing records** CMA.	CMA ≥80%	No	No	–
	Persistence	**Medication dispensing records.** Anniversary model	Persistent if you renew a prescription within a specific interval around the anniversary of your first prescription.			
Mortensen, J, 2008 (20)	Adherence	**Pill count.**	Adherent if no omitted doses or significant deviations.	No	–	–
		**Direct method:** thromboxane A2 (TxA2) synthesis in blood samples via ELISA.	Fully adherent if S-TxB2 levels were completely inhibited. Diagnostic threshold: CT (CT) <165 s using platelet function analyser (PFA-100) with collagen/epinephrine cartridges.	Comparison of PFA-100 with optical platelet aggregometry (OPA) across 4 days	PFA-100 reproducibility (CV): 9.9% (healthy) and 16.7% (patients). Correlation with OPA: r = 0.63 Sensitivity for aspirin non-response vs. OPA: 83%	
Newby KL, 2006 (81)	Adherence	**Self-report:** follow-up surveys about medication intake annually or every six months.	Adherent: If they reported the use of a medication in at least two consecutive surveys and continued to report it until the end of the study, their death, or withdrawal from follow-up.	No	–	–
O'Carroll R, 2011 (55)	Adherence	**Self-report:** MARS	High adherence: MARS scores close to 25.	Validated by other authors for patients with hypertension, asthma, and diabetes. (36)	Test-retest reliability: r = 0.75.	Cronbach's alpha: 0.68.
		**Direct method:** urinary salicylate level as an additional objective measure of adherence, calculated as the salicylate-to-creatinine ratio.	Diagnostic threshold for salicylate concentration	Authors provided evidence against validity of the method for 75 mg aspirin with single urine sample. The analytical method for the salicylate/creatinine ratio had been previously validated for salicylate detection in healthy adults, (89) although not specifically for adherence assessment, and the serum creatinine determination without protein precipitation was validated separately. (90)	No significant difference in salicylate levels between self-reported users and non-users (*p* = 0.13): the method lacked sensitivity to detect adherence	
Östbring MJ, 2021 (43)	Adherence	**Self-report:** MMAS-8.	MMAS-8 score (0 = high, 1–2 = medium, 3–6 = low adherence).	Validated by previous authors for hypertensive patients (results retracted) (26)	Sensitivity: 93%, Specificity: 53%.	Cronbach's alpha: 0.83.
	Persistence	**Medication dispensing records.**	Non-persistent if medication was not purchased 12–16 months post-discharge.	No	–	–
Rahhal, A, 2021 (82)	Adherence	**Medication dispensing records:** PDC.	PDC < 80%.	No	No	–
Rieckman, N, 2006 (83)	Adherence	**Electronic monitoring:** MEMS.	MEMS ≥ 80%	Validated by other authors for hypertensive patients. (39)	Sensitivity: 76%, Specificity: 83%, Correlation coefficient: 0.20.	–
Rieckman, N, 2006 (84)	Adherence	**Electronic monitoring:** MEMS.	Adherent: MEMS > 75%	Validated by other authors for hypertensive patients. (39)	Sensitivity: 76%, Specificity: 83%, Correlation coefficient: 0.20	–
Riegel, B, 2020 (85)	Adherence	**Electronic monitoring:** CleverCaps initially, later replaced by MEMS.	Adherence not explicitly defined; compares the number of days the medication bottle was opened (30, 60, or 90 days) between control and intervention groups.	Validated for hypertensive patients (39)	Sensitivity: 76%, Specificity: 83%.	Correlation coefficient: 0.20.
Riess H, 1986 (52)	Adherence	**Pill count.**	Adherent: 100%.	No	–	–
		**Direct method:** serum TxB2 levels via specific radioimmunoassay.	Non-adherent if TxB2 > 200 pg/ml	Demonstrated expected pharmacodynamic changes in TXB2 and aggregation post-aspirin; internal biological plausibility.	Significant drop in TXB2 only in aspirin group; aggregation and bleeding time supported pharmacodynamic effect. LOD: 50 pg/ml	–
Rinfret S, 2013 (45)	Adherence	**Self-report** method (simple question to the patient if they are taking ASA)	Adherent if answer Yes	No	–	–
		**Medication dispensing records**: PDC.	Adherent if PDC score ≥80%			
	Persistence	**Medication Dispensing Record**	Not defined			
		**Self-report method:** simple question to the patient	Persistent if answers YES Non-persistent: dispensing “gap” ≥14 days			
Robinson J.G., 2010 (46)	Adherence	**Pill count.**	Had taken >80% of pills over the last 28-day period.	No	–	–
	Persistence	**Self-report:** in-person interview about aspirin intake.	Reported medication use during two or more consecutive visits.			
Rubak P, 2013 (56)	Adherence	**Direct method:** plasma levels of ASA and SA using UHPLC.	Detection of ASA or SA in plasma.	Yes, analytical validation of UHPLC method.	Linearity (r² > 0.999) LOD: 0.170 µg/mL (ASA) and 0.053 µg/mL (SA) recovery: 89–103%. Intra-day precision (%RSD): ASA 1.3%–5.4%, SA 2.0%–6.3% Inter-day precision (%RSD): ASA 3.0%–7.5%, SA 4.2%–7.4%	–
Schwartz KA, 2005 (86)	Adherence	**Self-report:** oral interview about aspirin intake.	Adherent if confirmed appropriate medication intake.	No		–
		**Direct method:** aspirin inhibition of platelets (light aggregometry stimulated by arachidonic acid).	Adherent if platelet aggregation inhibition is observed following aspirin ingestion.	Before/after supervised aspirin, intake using aggregation test; internal validation through change in effect. Analytical method was validated for healthy adults by the same authors previously (54)	Threshold: aggregation slope reduction <50%. Concentration used: 125 µmol/L PPA (PPA). (54) Demonstrated reversal of apparent aspirin resistance after observed intake; aggregation normalized in most patients post-dose; functional validation through within-subject comparison.	
Shemesh E, 2006 (53)	Adherence	**Direct method:** platelet thromboxane B2 (TXB2) generation test (ELISA kit).	Adherent if TxB2 levels were consistent with the expected platelet inhibition after ASA intake. Non-adherence: TxB2 ≥ 12 ng/ml	Establishment of TXB2 cutoff from observed adherent patients; classification based on validated threshold. Analytical method was validated in previous studies for healthy adults (48)	Established TXB2 cutoff at 12 ng/mL based on supervised adherent patients (mean = 3.3 ng/mL, SD = 1.75); cutoff = mean + 5 SD; classified adherence based on this value	–
Simpson E, 2003 (40)	Adherence	**Medication dispensing records.**	Adherent if they have withdrawn the medication on a date close to the anniversary of your first prescription (days 305–365 of treatment).	No	No	–
	Persistence	**Medication dispensing records** PDC	PDC ≥ 80% during the first year.			
The Coronary Drug Project Research Group, 1976 (47)	Adherence	**Pill count**	Adherent if took 100% of pills.	Indirect evidence of validity	Urinary salicylate levels showed group separation and correlation with pill counts	–
		**Direct method:** salicylates in urine and serotonin release in platelets.	Biochemical evidence: aspirin absorbed and exerting effects on platelet function (via urinary salicylates and blood serotonin release).	No formal validation with sensitivity/specificity measures but authors provided indirect evidence for the use of salicylates in urine as adherence method. Serotonin release analytical method was validated in different study population by previous studies. (91)	Demonstrated discrimination between treated and untreated groups, temporal stability of test results.	–
	Persistence	Attendance at **follow-up visits** and treatment continuity.	Persistent if not explicitly defined.	No	–	–
Vora, P, 2022 (44)	Adherence	**Medication dispensing records:** MPR	Not specified	No	Sensitivity analysis conducted to determine the optimal cut-off point.	–
		**Medication dispensing records:** PDC	Not specified			
	Persistence	**Medication dispensing records.**	Absence of periods between the end of the medication and the supply of the next ≥ 60 days.			
Wei, L, 2008 (87)	Adherence	**Medication dispensing records:** PDC.	Good adherence ≥ 80% Partial adhesion: <80%	No	No	–
Wheat, H, 2021 (88)	Adherence	**Electronic monitoring:** MEMS.	Adherent if MEMS ≥ 80%	Validated for hypertensive patients) (39)	Sensitivity: 76%, Specificity: 83%, Correlation coefficient: 0.20.	–
Yasmina, A, 2017 (18)	Adherence	**Medication dispensing records**: PDC.	Not specified	No	No	–
	Persistence	**Medication dispensing records.**	Period between medication withdrawals ≤90 days, considered a “restarter” if the medication was withdrawn again after 90 days.			
Zhong J, 2023 (37)	Adherence	**Self-report:** questions about aspirin intake	Adherent if you take 100% tablets Lack of adherence if you take <100% Severe non-adherence if taking ≤75%	No	No	–
		**Pill count:** confirmed with a pillbox kit. Adherence was calculated by dividing the number of tablets taken by the total number of tablets that were to be taken in the specific period.				

AMI, acute myocardial infarction;, ASA, acetylsalicylic acid; CMA, continuous medication availability; CT, closure time; ELISA, enzyme-linked immunosorbent assay; LOD, limit of detection; MAQ, Morisky-Green medication adherence questionnaire; MARS, medication adherence report scale; MEMS, medication event monitoring system; MMAS-8, 8-item Morisky medication adherence scale; MPR, medication possession ratio; PDC, proportion of days covered; PFA-100, platelet function analyser-100; PPA, platelet prostaglandin agonist; S-TxB2, serum thromboxane B2; TxA2, thromboxane A2; TxB2, thromboxane B2; UHPLC, ultra-high performance liquid chromatography; AUC, area under the curve.

In addition to self-reported pill counts, some studies employed pill count methods performed by healthcare professionals. Two studies (20, 38) defined patients as adherent if the pill count indicated they followed the prescribed ASA regimen without missed doses or significant deviations. In one of these studies (38), adherence was further defined as pill counts ranging from 80% to 110% of the prescribed doses.

3. Medication dispensing records

Among dispensing record methods, PDC (*n* = 8), MPR (*n* = 3), continuous multiple-interval measure of medication availability (*n* = 1) (CMA) were the most utilized metrics, with adherence thresholds generally set at PDC, MPR or CMA ≥ 80%. The anniversary model was also used (*n* = 1)

4. Electronic monitoring methods

Several studies employed electronic devices to measure adherence to ASA. Typically, patients were considered adherent if they took at least 80% of the prescribed doses during the monitoring period. The devices used included: MEMS or Clever Caps (39). These devices electronically record each time the medication container is opened, providing accurate data on the frequency and timing of medication access.

#### Measurement of persistence

**Figure 3:**
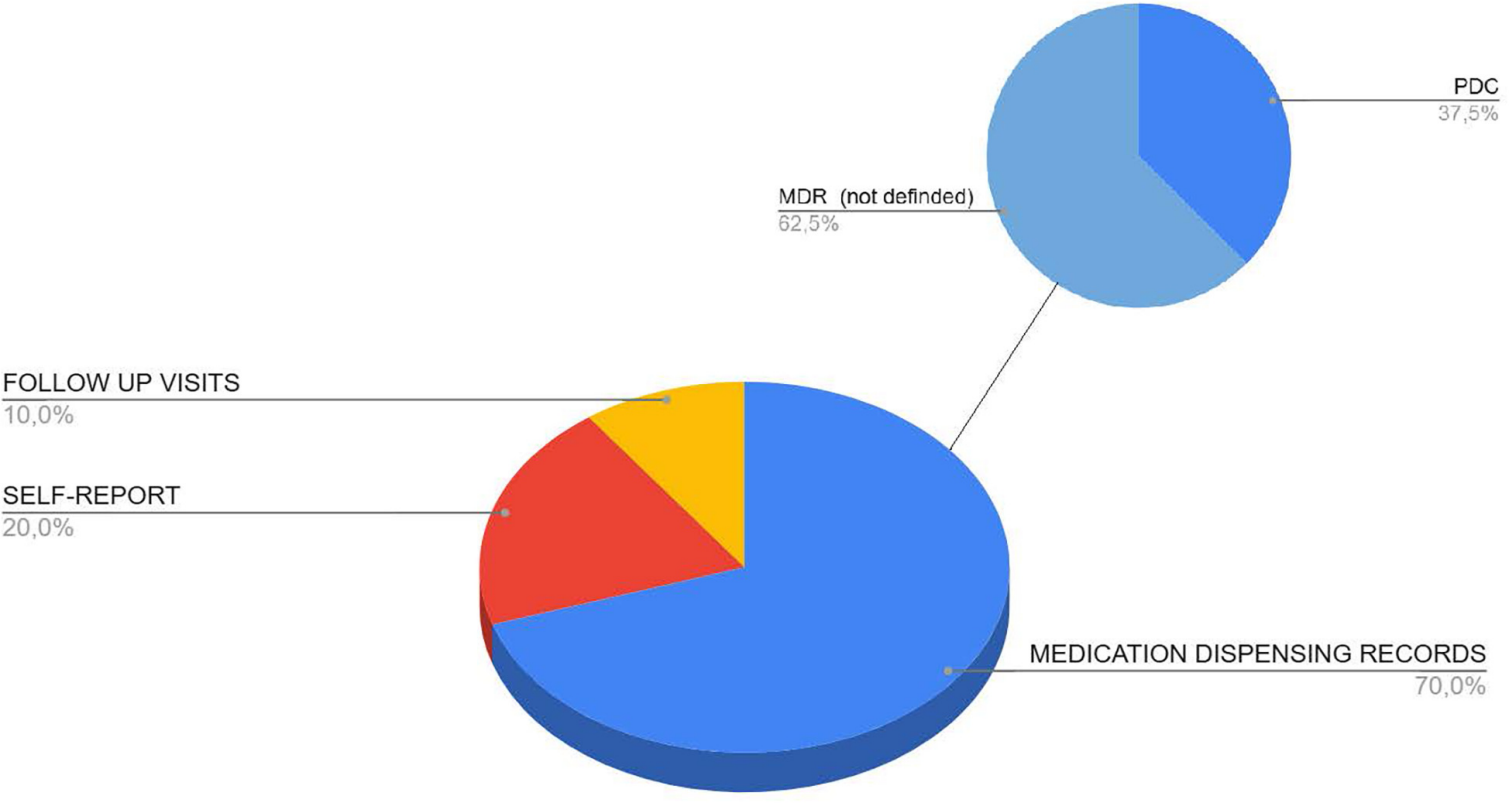
Pie chart illustrating the proportional distribution of aspirin persistence measurement methods reported in this review. MDR, Medication Dispensing Records; PDC, Proportion of Days Covered.

Table 2 shows the methods used to assess persistence with ASA in the reviewed studies. Persistence was evaluated using various indirect methods, with medication dispensing records being the most frequently employed approach, used in 70% (*n* = 7) of studies. Within this category, methods such as the PDC, the Anniversary Model, and Defined Gap Periods were commonly utilised. Self-report methods were less common, appearing in 30% (*n* = 2) of studies, and typically relied on patient interviews or follow-up visit attendance to determine persistence (*n* = 1). Figure 3 illustrates the percentage distribution of studies by the methods used to measure ASA persistence, highlighting the predominance of dispensing record-based approaches. The following sections describe each method in detail.

1. Medication dispensing records
  • PDC Record: One study (40) defined persistence as a PDC of 80% or higher during a one-year follow-up period. Other studies (33, 34) defined persistence based on dispensing interruptions, considering patients non-persistent if the gap exceeded 30 days. Another study (41) defined persistent patients if they continued their regimen without a gap of more than 28 days.
  • Anniversary model (42): Patients were classified as persistent if they dispenseded their prescription within a specific time frame surrounding the anniversary of their first prescription.
  • Defined gap periods (18, 43, 44): Persistence was measured by the absence of dispensing interruptions within specific periods, ranging from 60 to 90 days or within 12–16 months post-discharge.
2. Self-reported methods
  • Self-report interview (45, 46): Patients confirmed that they were taking their medication as prescribed.
3. Indirect follow-up or monitoring method
  • Follow-up visits and treatment continuity (47): Patients who attended follow-up visits and continued their treatment were considered persistent.

### Direct methods

#### Measurement of adherence

Several reviewed studies employed direct methods to assess adherence to ASA, focusing on its best-characterised effect: the irreversible inhibition of cyclooxygenase-1 (COX-1) in platelets, which reduces thromboxane A2 synthesis (48). These methods included:

• Platelet aggregation tests using ADP and collagen as agonists (20, 49): Patients were considered adherent if they demonstrated a sufficient reduction in platelet aggregation (typically below 60%).
• Thromboxane B2 (TxB2) measurement (50–53): serves as a biomarker for the inhibition of thromboxane A2 synthesis. In patients who are adherent to treatment, low levels of TxB2 in plasma indicate effective inhibition of platelet aggregation.
• Platelet Function Analyzer-100 (PFA-100) (20): This device measures primary hemostatic function by assessing closure time (CT) in whole blood. Patients with adhesion disorders typically exhibit low CT values (below 165 s).

Other tests included optical aggregation tests for arachidonic acid and ADP responses, where adherence was indicated by aggregation responses below specific thresholds (20, 54). Three studies utilised urine (47, 55) or plasma (56) tests to detect ASA or its metabolites. With regard to studies measuring the presence of AAS metabolites in urine, one study (47) complemented the detection of urinary salicylates with blood analysis to evaluate serotonin release by platelets when stimulated with epinephrine. Aspirin impacts this function by inhibiting thromboxane A2 synthesis in platelets, thereby impairing their ability to respond to such stimulation. Another study (55) assessed salicylic acid levels adjusted for creatinine in urine samples to account for urinary volume. The only study (56) measuring plasma concentrations of ASA and SA employed ultra-high-performance liquid chromatography (UHPLC).

### Validity indicators

None of the indirect methods used to measure adherence in the studies included in this review reported validity indicators specific to ASA adherence. Some methods, such as MEMS (39), the MMAS-8 (26) questionnaire (retracted), and the MMAS-4 (35), have validity indicators reported by previous authors, but these are for measuring adherence to other medications and in different populations. Table 2 presents the psychometric properties of these tools as reported in prior studies.

All direct methods are considered valid as they rely on established and validated analytical techniques to measure biological or chemical markers of ASA adherence. However, while some studies explicitly report validation data for the tests they employ, others do not provide this information. This inconsistency highlights the need for transparency in reporting validation processes to ensure reproducibility and reliability.

The methods used to measure ASA persistence lacked a formal validation process to ensure accuracy and consistency across different contexts. However, some authors conducted sensitivity analyses to justify the cut-off points for persistence, as seen in the study by Vora et al. (44), the sensitivity analysis confirmed that a 60-day cut-off for persistence and MPR calculations provided consistent estimates across different scenarios.

### Additional tools related to adherence prediction

Although not a method for measuring adherence or persistence, one study¹⁹ developed a nomogram designed to predict the risk of non-adherence to antiplatelet therapy in patients with congenital heart disease one year after discharge. The nomogram did not directly measure medication intake but instead predicted the likelihood of non-adherence based on patient behavior and clinical factors, using PDC as the gold standard for classification. Since this tool predicts the risk of non-adherence rather than measuring adherence behavior, it has been presented separately from the methods used to assess adherence or persistence. It represents a complementary approach focused on early identification of patients at high risk of non-adherence to guide preventive interventions.

### Risk of bias assessment

A risk of bias assessment was conducted for eight studies that provided empirical indicators of validity for methods used to assess adherence to aspirin, all of which involved direct measurement approaches such as platelet function tests or biochemical assays. The results of the RoB assessment, according to QUADAS-2 tool, are summarized in Table 3. Most studies were rated as having low risk of bias across all assessed domains. However, O'Carroll et al. (55) showed high risk in the index test and reference standard domains, due to the use of self-report as a comparator and the application of a urinary biomarker that failed to discriminate adherent from non-adherent patients. Shemesh et al. (53) was rated as moderate risk in patient selection due to limited information on recruitment methods. In several cases, the reference standard domain was marked as “not applicable”, as no external comparator was used.

**Table 3:** Risk of bias and applicability concerns assessed using the QUADAS-2 tool in studies reporting validity indicators of adherence measurement methods (*n* = 8).

Study	Risk of bias				Applicability concern		
	Domain 1 (Patient selection)	Domain 2 (Index test)	Domain 3 (Reference standard)	Domain 4 (Flow and timing)	Patient selection	Index test	Reference standard
Rubak P, 2013 (56)	Low	Low	Low	Low	Low	Low	Low
Mortensen J, 2008 (20)	Low	Low	Low	Low	Low	Low	Low
Komiya T, 1994 (49)	Low	Low	N/A	Low	Low	Low	N/A
Shemesh E, 2006 (53)	Moderate	Low	N/A	Low	Low	Low	N/A
Schwartz KA, 2005 (86)	Low	Low	N/A	Low	Low	Low	N/A
Riess H, 1986 (52)	Low	Low	N/A	Low	Low	Low	N/A
O'Carroll R, 2011 (55)	Low	High	High	Low	Low	High	High
Coronary Drug Project, 1976 (47)	Low	Low	N/A	Low	Low	Low	N/A

N/A, not applicable.

It should be acknowledged that only five of the included studies (20, 47, 53, 56) were specifically designed to validate aspirin adherence methods, using approaches such as comparison with established reference standards (e.g., UHPLC, PFA-100, or direct observation) or by defining biological cut-off points. The remaining three studies (49, 52, 55) were not designed as validation studies, although some provided indirect evidence on the usefulness or limitations of the methods applied, for example, O'Carroll et al. (55) found that urinary salicylate levels did not effectively distinguish between adherent and non-adherent patients.

## Discussion

The results of this systematic review show that the methods used to measure adherence to ASA in SCP populations are primarily indirect. These methods include self-report techniques, such as questionnaires like MMAS-8 (26), MMAS-4 (35), and MARS (36), as well as standardised telephone or in-person surveys and self-report forms. Prescription and medication supply record reviews, mainly PDC and MPR, are also widely used, alongside electronic monitoring (e.g., MEMS), pill count methods, and direct methods measuring platelet activity or ASA/metabolite detection in blood or urine. In terms of persistence, most studies relied on medication dispensing records, particularly PDC and the anniversary model. Regarding the validity indicators of the methods used, none of the indirect methods had specific validation for measuring ASA adherence (or persistence).

### Terminology

Terminology related to adherence and persistence is inconsistently used in the literature. Therefore, this review considered the terminology used in different studies (“adherence” or “persistence”) regardless of whether it strictly followed the definitions (57). Many studies use these terms interchangeably, even though adherence refers to the proportion of prescribed doses taken, while persistence refers to the continuous use of treatment without interruption. Additionally, terms like “compliance” and “concordance” have been used to define various aspects of medication use. However, “compliance” often carries a negative connotation of subordination to the prescriber (58, 59), and “concordance” is frequently misunderstood as synonymous with compliance (60–62). These inconsistencies hinder cross-study comparisons and make it difficult to draw definitive conclusions about the effectiveness of interventions aimed at improving adherence. Standardising terminology and measurement methods is essential to improve comparability and support evidence-based decision-making.

### Indirect methods

Dispensing records are among the most widely used approaches for evaluating adherence and persistence. The most frequently used methods are PDC, followed by MPR and the anniversary model, with their evaluation often subject to the interpretation of study authors. PDC calculates the percentage of days a patient has the medication available during a given period, excluding oversupply from early dispensations. This metric is robust and widely accepted due to its consistency and positive association with clinical outcomes (63). In contrast, MPR calculates the proportion of time a patient has medication available but can exceed 100%, indicating oversupply. The main limitation of these methods is that they do not confirm whether the patient actually takes the medication. In this regard, Cao et al.'s predictive nomogram (19) demonstrates how these limitations can be overcome by integrating multiple clinical and social risk factors, allowing not only the measurement of adherence using PDC but also the prediction of the risk of non-adherence. This highlights the importance of combining quantitative metrics with predictive tools to design proactive intervention strategies aimed at evaluating and improving actual treatment adherence. Persistence is generally measured through dispensing records, particularly the PDC. Although PDC provides a general idea of adherence, it is not ideal for measuring persistence, as it does not guarantee continuous treatment without interruptions. Some studies have adapted PDC to measure persistence using interruption criteria (≥30, ≥60, or ≥90 days).

Self-report methods are simple and practical tools for evaluating adherence from the patient's perspective. However, their validity may be affected by recall bias or social desirability (64). The MMAS-8 (26) and its previous version, MMAS-4 (35), are among the most widely used questionnaires for chronic diseases, although they were originally developed and validated specifically for patients with hypertension. These questionnaires have been studied in numerous populations and contexts, yielding varied psychometric results. In some studies, the MMAS-8 has demonstrated good validity and reliability (27, 29), while in others, its internal consistency and predictive capacity have been limited (30), suggesting that its accuracy may depend on the context and specific population. Notably, the original Morisky study, which developed and validated the MMAS-8, has been retracted, raising questions about the instrument's validity and the integrity of its psychometric properties (26). This retraction, combined with the variability in performance across different patient populations, highlights significant limitations in the use of the MMAS-8 (26) and MMAS-4 (35) as adherence measurement tools. In light of these issues, alternative self-report instruments with stronger psychometric support, such as the MARS (36), should be considered. The MARS has demonstrated more consistent reliability and validity across various clinical contexts and has not been affected by concerns of scientific misconduct. Future research efforts should prioritize the use and further validation of such tools to ensure more accurate and trustworthy adherence assessments.

Telephone or in-person questionnaires are considered practical tools for assessing adherence. The direct patient-reported adherence assessment via medication recall and confirmation, in person or by phone, where patients are asked to list their medications and are considered adherent if their answers match the prescribed medications at discharge or are directly asked whether they are taking their medications as instructed, is widely used among the self-reported methods. However, their validity can be limited by reliance on patient honesty and accuracy, introducing potential information bias that could affect the accuracy of adherence measurements. Pill count through self-report is an indirect method used in some adherence studies, although its application in ASA evaluation is limited. It involves counting leftover pills in a container to infer adherence. While it is cost-effective, its validity is limited as it does not guarantee that the patient has taken the recorded doses.

Electronic monitoring devices like MEMS are regarded as a reference method for evaluating adherence, as they provide a detailed record of patient behavior. However, their validity is limited because they do not confirm whether the patient actually ingests the medication after opening the container. While they are useful approximations, they have significant limitations and are not recommended as the sole reference for assessing adherence (65).

### Direct methods

Unlike other drugs, such as statins, where their concentration or that of their metabolites is measured in plasma or urine, (66) in the case of aspirin, due to its short half-life, direct methods instead evaluate its pharmacodynamic effect: the irreversible inhibition of platelet cyclooxygenase-1, which in turn inhibits thromboxane A2 synthesis. These methods aim to capture the functional consequences of this mechanism, including optical platelet aggregation tests, plasma thromboxane B2 (TxB2) measurement, PFA-100 tests, ADP and collagen-induced aggregation, and serotonin release induced by epinephrine in platelets (48). They offer an objective and personalised assessment of treatment effectiveness, helping to identify individual responses and detect adherence or resistance issues. However, most direct methods require invasive biological samples, which limits their routine clinical applicability. Moreover, costs and availability restrict their large-scale use outside of research or specific clinical settings.

Measurements of platelet aggregation effects in ASA-treated patients present various limitations, such as strict adherence thresholds that may misclassify patients. Biological variability, aspirin resistance, concurrent medications, health conditions, and irregular aspirin intake can all affect the results, complicating interpretation (20, 49, 67, 68). Cotter et al.'s study (50) suggests that many cases classified as biologically resistant to aspirin are actually due to non-adherence, highlighting the importance of assessing adherence before assuming biological resistance.

Three reviewed studies (47, 55, 56) utilised urine or plasma ASA/metabolite detection. One study (47) complemented salicylate detection with epinephrine-induced platelet serotonin release to assess platelet function. Urinary salicylate levels reflect recent aspirin intake but have limitations due to the short half-life of salicylates (69). Platelet function tests, however, provide a more enduring indicator of aspirin's therapeutic effect. In another study (55), salicylic acid levels adjusted for creatinine were measured in urine samples to control for urine volume. This method was ineffective at distinguishing between patients taking aspirin and those not taking it, likely due to aspirin's short half-life and low doses (75 mg). The only study (56) measuring plasma ASA and SA concentrations using UHPLC concluded that SA is a more sensitive marker due to its stability and longer half-life, but it also presents limitations, such as high interindividual variability and the risk of becoming undetectable after 24 hours at low doses.

### Strengths and limitations of direct vs. indirect methods

Although both direct and indirect methods have been used to measure adherence, their applicability varies significantly depending on the context. Direct methods, such as platelet function tests and thromboxane B2 assays, offer objective measures of aspirin intake (adherence) but are costly, invasive, and require specialized laboratory infrastructure, limiting their use to research settings or specialized clinics. In contrast, indirect methods, such as dispensing records and self-reports, are used to assess both adherence and persistence, and are more feasible and widely adopted in routine clinical practice and large-scale studies, despite their susceptibility to information and recall bias. In settings with integrated electronic health systems, such as the United States, dispensing records are the predominant method due to their accessibility and linkage to clinical outcomes. In outpatient and resource-limited contexts, self-report questionnaires remain more common due to their low cost and ease of administration. Thus, the choice of measurement method is strongly influenced by feasibility, available infrastructure, and the specific objectives of each study.

Despite their widespread use, indirect methods such as dispensing records and self-reports have not been specifically validated for measuring adherence or persistence to aspirin, reflecting both pharmacological and methodological challenges. Ideally, validation would involve comparison against a gold standard such as the direct measurement of drug or metabolite levels. However, aspirin's short half-life, rapid metabolism into salicylate, and fast elimination, especially at low doses, make biochemical detection unreliable. Moreover, the pharmacodynamic effect of aspirin persists longer than its biochemical detectability, complicating direct validation. Although electronic monitoring systems (e.g., MEMS) could offer an alternative objective reference, no studies have systematically validated dispensing records for aspirin adherence using such technologies. This gap highlights the need for standardized validation protocols that combine dispensing data with objective measurements adapted to aspirin's pharmacological properties.

### Validation gaps and methodological challenges

Although the number of studies providing validation data for adherence measurement methods was limited, the overall risk of bias among these studies was low. Most studies employed direct measurement techniques and were judged to have low risk of bias across the main QUADAS-2 domains. However, some concerns emerged, particularly regarding the absence of an independent reference standard in several studies and a high risk of bias in one study using a non-validated biomarker. These limitations highlight the need for more methodologically robust validation studies that include appropriate comparators and standardized evaluation criteria.

This study highlights the lack of validated ASA adherence measurement methods for SCP, emphasizing the urgent need to develop clinically applicable tools that incorporate gender perspectives. Although gender is a known independent predictor of non-adherence (70), most studies overlook gender disparities in adherence measurement. For example, women are 25% less likely to adhere to combined medication regimens after a myocardial infarction compared to men (71). Despite the critical influence of gender on adherence behaviors, none of the adherence measurement methods identified in this review explicitly accounted for gender differences in their design, validation, or application.

Current indirect methods, such as dispensing records and self-report questionnaires, typically fail to capture gender-specific barriers, including caregiving responsibilities, differential access to healthcare, and variations in perceptions of medication necessity. To improve the accuracy and equity of adherence measurement, future development of ASA adherence tools should combine dispensing records, self-reports, and direct methods, validated through comprehensive psychometric testing and explicitly incorporating gender-sensitive items and analytical strategies.

### Proposed characteristics for optimal adherence and persistence assessment methods

To improve clinical management, methods for assessing adherence and persistence should meet several key criteria. They should be valid and reliable, ideally demonstrating high sensitivity and specificity to accurately distinguish between adherent and non-adherent patients. Feasibility is also essential: methods should be non-invasive, cost-effective, and easily applicable in routine clinical settings without imposing a significant burden on patients or healthcare providers. Additionally, they should be capable of assessing both adherence (execution of dosing) and persistence (continuation of therapy over time) separately, to provide a comprehensive picture of medication-taking behaviors.

Moreover, optimal methods should incorporate a patient-centered approach, considering individual barriers to adherence, including those related to gender, socioeconomic status, and healthcare access. Tools combining multiple data sources, such as pharmacy records, electronic monitoring, and validated self-report questionnaires, would offer a more accurate and holistic assessment. The development of predictive tools, capable of identifying patients at high risk of non-adherence before clinical deterioration occurs, should also be prioritized. These features would enhance the ability to implement timely and tailored interventions, ultimately improving treatment outcomes in secondary cardiovascular prevention.

### Study limitations

This systematic review has some limitations. We restricted the search to studies published in languages using Latin alphabets, which likely had a minimal impact on the findings, as the majority of relevant studies were published in English. Additionally, the search was conducted in three major databases (EMBASE, MEDLINE, and Scopus), and grey literature as well as studies indexed exclusively in other sources were not included. Consequently, there is a possibility that some relevant studies may have been missed, although we believe this risk is limited given the broad coverage of the selected databases. Lastly, two of the reviewers involved in study selection were student researchers; however, their work was conducted under the supervision of experienced researchers, and all screenings were performed independently and verified through conflict resolution procedures to ensure the accuracy and consistency of the selection process.

## Conclusions

The methods used to measure adherence to ASA in secondary cardiovascular prevention were primarily indirect, relying on the review of prescription and supply records, self-reporting methods, and electronic monitoring. To a lesser extent, pill count methods and direct approaches, such as blood-based platelet function tests—specifically platelet aggregation—were also employed. For measuring persistence, the methods utilised were based on medication dispensing records. Most indirect methods lacked specific validation indicators for measuring adherence to aspirin, highlighting the need for standardization and validation efforts tailored to this context.

## Data Availability

The original contributions presented in the study are included in the article/Supplementary Material, further inquiries can be directed to the corresponding author.
