# Supplementary material for: Methods and validity indicators for measuring adherence and persistence to aspirin in secondary cardiovascular prevention: a systematic review
Source: Front Cardiovasc Med. 2025 May 26;12:1570331. doi: 10.3389/fcvm.2025.1570331 (PMC12146176; doi:10.3389/fcvm.2025.1570331)
Supplement: Supplementary file 1 [file Datasheet1.pdf]

Supplementary Material 1

**Keywords**	**Type**
Cardiovascular diseases	MESH
Cardiovascular disease	Free text term
Ischemic heart disease	Free text term
Myocardial ischemia	MESH
Myocardial ischemia	Free text term
Angina	Free text term
Angina Pectoris	MESH
Diabetic retinopathy	MESH
Diabetic retinopathy	Free text term
Hypertensive retinopathy	MESH
Hypertensive retinopathy	Free text term
Aortic Aneurysm	MESH
Aortic Aneurysm	Free text term
Aortic dissection	MESH
Aortic dissection	Free text term
Myocardial Infarction	MESH

Myocardial Infarction	Free text term
Stroke	Free text term
Stroke	MESH
Hemorrhagic stroke	MESH
Embolic stroke	MESH
Ischemic stroke	MESH
Thrombotic stroke	MESH
Brain attack	Free text term
Transient brain accident	Free text term
Renal Insufficiency	MESH
Chronic Renal Insufficiency	MESH
Renal Insufficiency	Free text term
Kidney diseases	MESH
Renal disease	Free text term
Kidney injury	Free text term
Kidney failure	Free text term
Acute kidney injury	MESH
Chronic kidney failure	MESH
Heart Failure	MESH
Heart Failure	Free text term

Peripheral Arterial Disease	MESH
Peripheral Arterial Disease	Free text term
Peripheral vascular Disease	MESH
Peripheral vascular Disease	Free text term
Lower limb arterial disease	Free text term
Diabetic macular edema	Free text term
Acetylsalicylic acid	Free text term
Aspirin	MESH
Aspirin	Free text term
Ischemic attack, transient	MESH
Secondary cardiovascular prevention	MESH
Heart attack	Free text term
Coronary artery disease	Free text term
Coronary artery disease	MESH
Coronary disease	MESH
Coronary heart disease	Free text term
Acute coronary syndrome	MESH
Acute coronary syndrome	Free text term
Treatment Adherence and Compliance	MESH
Compliance	Free text term
Medication Adherence	MESH

Adherence Methods	Free text term
Patient compliance	MESH
Adher*	Free text term
Nonadher*	Free text term
Noncompliance	Free text term
Incompliance	Free text term
Non-adherence	Free text term
Non adherence	Free text term
Nonadherent	Free text term
Non adherent	Free text term
Non-persistence	Free text term
Non persistence	Free text term
Non-persistent	Free text term
Non-compliance	Free text term
Non compliance	Free text term
Cooperation	Free text term
Therapeutic adhesion	Free text term

Search strings

Database: MEDLINE

Search date: October 30 2023

#1

(((Cardiovascular diseases[MeSH Terms]) OR (Myocardial ischemia[MeSH Terms])) OR (angina pectoris[MeSH Terms])) OR (diabetic retinopathy[MeSH Terms])) OR (hypertensive retinopathy[MeSH Terms])) OR (aortic aneurysm[MeSH Terms])) OR (Aortic dissection[MeSH Terms])) OR (Myocardial Infarction[MeSH Terms])) OR (Stroke[MeSH Terms])) OR (Hemorrhagic stroke[MeSH Terms])) OR (Embolic stroke[MeSH Terms])) OR (Ischemic stroke[MeSH Terms])) OR (Thrombotic stroke[MeSH Terms])) OR (Renal Insufficiency[MeSH Terms])) OR (Renal Insufficiency, chronic[MeSH Terms])) OR (Kidney diseases[MeSH Terms])) OR (Acute kidney injury[MeSH Terms])) OR (Kidney failure, chronic[MeSH Terms])) OR (Heart Failure[MeSH Terms])) OR (Peripheral Arterial Disease[MeSH Terms])) OR (Peripheral vascular Disease[MeSH Terms])) OR (Ischemic attack, transient[MeSH Terms])) OR (Secondary cardiovascular prevention[MeSH Terms])) OR (Acute coronary syndrome[MeSH Terms])) OR (Coronary artery disease[MeSH Terms])) OR (Coronary disease[MeSH Terms])) OR (Acute coronary syndrome[MeSH Terms])) OR ("Cardiovascular disease"[Title/Abstract])) OR ("Ischemic heart disease"[Title/Abstract])) OR ("Myocardial ischemia"[Title/Abstract])) OR ("Angina"[Title/Abstract])) OR ("Diabetic retinopathy"[Title/Abstract])) OR ("Hypertensive retinopathy"[Title/Abstract])) OR ("Aortic Aneurysm"[Title/Abstract])) OR ("Aortic dissection"[Title/Abstract])) OR ("Myocardial Infarction"[Title/Abstract])) OR (stroke[Title/Abstract])) OR ("Brain attack"[Title/Abstract])) OR ("Transient brain accident"[Title/Abstract])) OR ("Renal Insufficiency"[Title/Abstract])) OR ("Renal

disease"[Title/Abstract])) OR ("Kidney injury"[Title/Abstract])) OR ("Kidney failure"[Title/Abstract])) OR ("Heart Failure"[Title/Abstract])) OR ("Peripheral Arterial Disease"[Title/Abstract])) OR ("Peripheral vascular Disease"[Title/Abstract])) OR ("Lower limb arterial disease"[Title/Abstract])) OR ("Diabetic macular edema"[Title/Abstract])) OR ("Secondary cardiovascular prevention"[Title/Abstract])) OR (ACS[Title/Abstract])) OR ("Acute coronary syndrome"[Title/Abstract])) OR ("Heart attack"[Title/Abstract])) OR ("Coronary artery disease"[Title/Abstract])) OR ("Coronary heart disease"[Title/Abstract])

N= 3,534,752

#2

((Aspirin[MeSH Terms]) OR (Aspirin[Title/Abstract])) OR ("Acetylsalicylic acid"[Title/Abstract])

N= 77,729

#3

((((((((((((((((((Adher*[Title/Abstract]) OR (Treatment Adherence and Compliance[MeSH Terms])) OR ("Medication Adherence"[MeSH Terms])) OR ("Patient compliance"[MeSH Terms])) OR ("Compliance"[Title/Abstract])) OR ("Adherence Methods"[Title/Abstract])) OR ("Noncompliance"[Title/Abstract])) OR ("Incompliance"[Title/Abstract])) OR ("Non-adherence"[Title/Abstract])) OR ("Non adherence"[Title/Abstract])) OR (Nonadherent[Title/Abstract])) OR (Cooperation[Title/Abstract])) OR ("Non adherent"[Title/Abstract])) OR ("Non-persistence"[Title/Abstract])) OR ("Non persistence"[Title/Abstract])) OR ("Non-persistent"[Title/Abstract])) OR ("Non-

compliance"[Title/Abstract])) OR ("Non compliance"[Title/Abstract])) OR ("Therapeutic adhesion"[Title/Abstract])

N= 673,581

#1 AND #2 AND #3

N= 1,596

Filters:

(address[Filter] OR autobiography[Filter] OR bibliography[Filter] OR biography[Filter] OR booksdocs[Filter] OR casereports[Filter] OR clinicalconference[Filter] OR clinicaltrialprotocol[Filter] OR veterinaryclinicaltrial[Filter] OR comment[Filter] OR congress[Filter] OR consensusdevelopmentconference[Filter] OR consensusdevelopmentconferencenih[Filter] OR dictionary[Filter] OR directory[Filter] OR editorial[Filter] OR electronicsupplementarymaterials[Filter] OR englishabstract[Filter] OR governmentpublication[Filter] OR guideline[Filter] OR interactivetutorial[Filter] OR interview[Filter] OR introductoryjournalarticle[Filter] OR lecture[Filter] OR legalcase[Filter] OR legislation[Filter] OR letter[Filter] OR meta-analysis[Filter] OR news[Filter] OR newspaperarticle[Filter] OR veterinaryobservationalstudy[Filter] OR patienteducationhandout[Filter] OR periodicalindex[Filter] OR personalnarrative[Filter] OR portrait[Filter] OR practiceguideline[Filter] OR review[Filter] OR scientificintegrityreview[Filter] OR systematicreview[Filter] OR technicalreport[Filter] OR videoaudiomedia[Filter] OR webcast[Filter])

Filtro: Humans

N=1022

Database: EMBASE

Search date: October 30, 2023

#1

'cardiovascular disease':ti,ab OR 'cardiovascular disease'/mj/exp OR 'cardiovascular disease' OR 'ischemic heart disease':ab,ti OR 'heart muscle ischemia'/mj/exp OR 'heart muscle ischemia' OR 'myocardial ischemia':ab,ti OR 'angina':ab,ti OR 'angina pectoris'/mj/exp OR 'angina pectoris' OR 'diabetic retinopathy'/mj/exp OR 'diabetic retinopathy' OR 'diabetic retinopathy':ab,ti OR 'hypertension retinopathy'/mj/exp OR 'hypertension retinopathy' OR 'hypertensive retinopathy':ab,ti OR 'aortic aneurysm'/mj/exp OR 'aortic aneurysm' OR 'aortic aneurysm':ab,ti OR 'aortic dissection'/mj/exp OR 'aortic dissection' OR 'aortic dissection':ab,ti OR 'myocardial infarction':ab,ti OR stroke:ab,ti OR 'hemorrhagic stroke':ab,ti OR 'cardioembolic stroke'/mj/exp OR 'cardioembolic stroke' OR 'embolic stroke':ab,ti OR 'ischemic stroke'/mj/exp OR 'ischemic stroke' OR 'ischemic stroke':ab,ti OR 'thrombotic stroke':ab,ti OR 'cerebrovascular accident'/mj/exp OR 'cerebrovascular accident' OR 'brain attack':ab,ti OR 'transient brain accident':ab,ti OR 'renal insufficiency, chronic':ab,ti OR 'renal insufficiency':ab,ti OR 'kidney disease'/mj/exp OR 'kidney disease' OR 'kidney diseases':ab,ti OR 'kidney injury'/mj/exp OR 'kidney injury' OR 'kidney injury':ab,ti OR 'kidney failure'/mj/exp OR 'kidney failure' OR 'kidney failure':ab,ti OR 'acute kidney failure'/mj/exp OR 'acute kidney failure' OR 'acute kidney injury':ab,ti OR 'chronic kidney failure'/mj/exp OR 'chronic kidney failure' OR 'kidney failure, chronic':ab,ti OR 'heart failure'/mj/exp OR 'heart failure' OR 'heart failure':ab,ti OR 'peripheral arterial disease'/mj/exp OR 'peripheral arterial disease' OR

'peripheral arterial disease':ab,ti OR 'peripheral vascular disease'/mj/exp OR 'peripheral vascular disease'/exp OR 'peripheral vascular disease' OR 'lower limb arterial disease':ab,ti OR 'diabetic macular edema'/mj/exp OR 'diabetic macular edema'/exp OR 'diabetic macular edema' OR 'transient ischemic attack'/mj/exp OR 'transient ischemic attack' OR 'ischemic attack, transient':ab,ti OR 'secondary cardiovascular prevention':ab,ti OR 'heart infarction'/mj/exp OR 'heart infarction' OR 'heart attack':ab,ti OR 'coronary artery disease'/mj/exp OR 'coronary artery disease' OR 'coronary artery disease':ab,ti OR 'coronary disease':ab,ti OR 'ischemic heart disease'/mj/exp OR 'ischemic heart disease' OR 'coronary heart disease':ab,ti OR 'acute coronary syndrome'/mj/exp OR 'acute coronary syndrome' OR 'acute coronary syndrome':ab,ti

N= 5559707

#2

'acetylsalicylic acid'/mj OR 'acetylsalicylic acid':ab,ti OR aspirin:ab,ti

N=123568

#3

'patient compliance'/mj OR adherence:ab,ti OR compliance:ab,ti OR adher*:ab,ti OR incompliance:ab,ti OR 'non adherence':ab,ti OR persistence:ab,ti OR 'non persistence':ab,ti OR 'therapeutic adhesion':ti,ab OR 'non-compliance':ti,ab OR 'noncompliance':ti,ab OR 'cooperation':ti,ab OR 'non-persistent':ab,ti

N= 818,217

#1 AND #2 AND #3

N= 2899

Filters:

Included:

('article'/it OR 'article in press'/it)

([adult]/lim OR [aged]/lim OR [middle aged]/lim OR [very elderly]/lim OR [young adult]/lim)

[embase]/lim

Excluded:

('animal experiment'/de OR 'animal model'/de OR 'case report'/de OR 'consensus development'/de OR 'evidence based practice'/de OR 'human cell'/de OR 'human tissue'/de OR 'in vitro study'/de OR 'interview'/de OR 'meta analysis'/de OR 'nonhuman'/de OR 'practice guideline'/de OR 'systematic review'/de)

N= 85

Database: SCOPUS

Search date: October 30, 2023

(TITLE-ABS-KEY("Cardiovascular disease") OR TITLE-ABS-KEY("Ischemic heart disease") OR TITLE-ABS-KEY("Myocardial ischemia") OR TITLE-ABS-KEY(Angina) OR TITLE-ABS-KEY("Angina Pectoris") OR TITLE-ABS-KEY("Diabetic retinopathy")OR TITLE-ABS-KEY("Hypertensive retinopathy") OR TITLE-ABS-KEY("Hypertensive retinopathy") OR TITLE-

ABS-KEY("Aortic Aneurysm") OR TITLE-ABS-KEY("Aortic dissection") OR TITLE-ABS-KEY("Myocardial Infarction") OR TITLE-ABS-KEY("Stroke") OR TITLE-ABS-KEY("Hemorrhagic stroke") OR TITLE-ABS-KEY("Embolic stroke") OR TITLE-ABS-KEY("Ischemic stroke") OR TITLE-ABS-KEY("Thrombotic stroke") OR TITLE-ABS-KEY("Brain attack") OR TITLE-ABS-KEY("Transient brain accident") OR TITLE-ABS-KEY("Renal Insufficiency") OR TITLE-ABS-KEY("Renal Insufficiency, chronic") OR TITLE-ABS-KEY("Kidney diseases") OR TITLE-ABS-KEY("Renal disease") OR TITLE-ABS-KEY("Kidney injury") OR TITLE-ABS-KEY("Kidney failure") OR TITLE-ABS-KEY("Acute kidney injury") OR TITLE-ABS-KEY("Kidney failure, chronic") OR TITLE-ABS-KEY("Heart Failure") OR TITLE-ABS-KEY("Peripheral Arterial Disease") OR TITLE-ABS-KEY("Peripheral vascular Disease") OR TITLE-ABS-KEY("Lower limb arterial disease") OR TITLE-ABS-KEY("Diabetic macular edema") OR TITLE-ABS-KEY("Ischemic attack, transient") OR TITLE-ABS-KEY("Secondary cardiovascular prevention") OR TITLE-ABS-KEY("Heart attack") OR TITLE-ABS-KEY("Coronary artery disease") OR TITLE-ABS-KEY("Coronary disease") OR TITLE-ABS-KEY("Coronary heart disease") OR TITLE-ABS-KEY("Acute coronary syndrome")) AND (TITLE-ABS-KEY("aspirin") OR TITLE-ABS-KEY("Acetylsalicylic acid")) AND (TITLE-ABS-KEY("treatment adherence") OR TITLE-ABS-KEY("medication adherence") OR TITLE-ABS-KEY("therapeutic adherence") OR TITLE-ABS-KEY("therapy adherence")OR TITLE-ABS-KEY("patient compliance") OR TITLE-ABS-KEY("medication persistence") OR TITLE-ABS-KEY("medication compliance") OR TITLE-ABS-KEY("therapeutic adhesion") OR TITLE-ABS-KEY("patient persistence") OR TITLE-ABS-KEY("incompliance")) AND (EXCLUDE (SUBJAREA,"ENVI") OR EXCLUDE (SUBJAREA,"DENT") OR EXCLUDE (SUBJAREA,"ARTS") OR EXCLUDE (SUBJAREA,"MATE")) AND (LIMIT-TO (DOCTYPE,"ar")) AND (EXCLUDE (LANGUAGE,"Russian") OR EXCLUDE (LANGUAGE,"Chinese") OR EXCLUDE (

LANGUAGE,"Korean") OR EXCLUDE (LANGUAGE,"Japanese") OR EXCLUDE (LANGUAGE,"Arabic")) AND (LIMIT-TO (EXACTKEYWORD,"Adult"))

N= 1184
